# Perspectives on Assembling Coronavirus Spikes on Fiber Optics to Reveal Broadly Recognizing Antibodies and Generate a Universal Coronavirus Detector

**DOI:** 10.3389/fbioe.2021.637715

**Published:** 2021-11-26

**Authors:** Marzhan Sypabekova, Daniele Tosi, Luca Vangelista

**Affiliations:** ^1^ School of Medicine, Nazarbayev University, Nur-Sultan, Kazakhstan; ^2^ School of Engineering and Digital Sciences, Nazarbayev University, Nur-Sultan, Kazakhstan; ^3^ Laboratory of Biosensors and Bioinstruments, National Laboratory Astana, Nur-Sultan, Kazakhstan

**Keywords:** COVID-19, coronavirus, spike, broadly neutralizing antibodies, biosensor, optical fiber

## Abstract

In time of COVID-19 biological detection technologies are of crucial relevance. We propose here the use of state of the art optical fiber biosensors to address two aspects of the fight against SARS-CoV-2 and other pandemic human coronaviruses (HCoVs). Fiber optic biosensors functionalized with HCoV spikes could be used to discover broadly neutralizing antibodies (bnAbs) effective against known HCoVs (SARS-CoV, MERS-CoV and SARS-CoV-2) and likely future ones. In turn, identified bnAbs, once immobilized onto fiber optic biosensors, should be capable to detect HCoVs as diagnostic and environmental sensing devices. The therapeutic and preventative value of bnAbs is immense as they can be used for passive immunization and for the educated development of a universal vaccine (active immunization). Hence, HCoV bnAbs represent an extremely important resource for future preparedness against coronavirus-borne pandemics. Furthermore, the assembly of bnAb-based biosensors constitutes an innovative approach to counteract public health threats, as it bears diagnostic competence additional to environmental detection of a range of pandemic strains. This concept can be extended to different pandemic viruses, as well as bio-warfare threats that entail existing, emerging and extinct viruses (e.g., the smallpox-causing *Variola virus*). We report here the forefront fiber optic biosensor technology that could be implemented to achieve these aims.

## Introduction

Biosensors play an important role in the detection of viruses, diagnosis of viral infection and possibly prevention of viral pandemics (Taha et al., 2020). In the planetary efforts to fight COVID-19, technological advancement proceeded at unprecedented speed and scientists’ creativity has rarely been so fertile. Within this outlook, we propose the development of a combined set of technologies, in the attempt to discover broadly neutralizing antibodies (bnAbs) directed against the spike of human coronaviruses (HCoVs) and, in turn, embed them in fiber optic biosensors to detect HCoVs. BnAbs are protective antibodies present in the blood of individuals that won the fight against SARS-CoV-2 and considered to provide protection against a large number of viral strains (Vangelista and Secchi, 2020). Current biosensing methods for COVID-19 (Choi, 2020; Ravi et al., 2020; Santiago, 2020), make use of receptor binding domain (RBD)-specific antibodies to detect SARS-CoV-2 and stand out as alternative methods for rapid testing. However, several SARS-CoV-2 emerging variants present mutations in the RBD which could alter the binding (Sui et al., 2014) and compromise the efficacy of these biosensors. Conventional technologies such as quantitative real time polymerase chain reaction (qRT-PCR) have been broadly used to detect COVID-19, however they are time-consuming, labor-intensive, require trained personnel and are unavailable in remote settings. Fiber optic biosensors offer a real-time detection, in air or in liquid; extremely low limit of detection; remote sensing (fiber can sense in a “contaminated” environment, interrogator placed far away). Also, as antibodies are small compared to the wavelength, the biological system on the surface and the fiber sensing system itself are almost perfectly decoupled. The spike protein is responsible for HCoV receptor-mediated tropism and viral membrane fusion with target cell membrane. The spike is the major target of infected individuals’ antibody response and by far the major antigen used in vaccine development. Neutralizing antibodies effective against SARS-CoV and SARS-CoV-2 spikes have been discovered (Pinto et al., 2020; Yuan et al., 2020) and recent studies reported the importance and potential use of bnAbs in therapeutics (Cully, 2021; Garcia-Beltran et al., 2021). After decades of search, the fight against different pandemic viruses, such as HIV-1, influenza and Ebola virus, is now being empowered enormously by the implementation of rare bnAbs isolated from survivors presenting exceptional immune responses (Vangelista and Secchi, 2020). The encounter of humanity with pandemic HCoVs such as SARS-CoV, MERS-CoV and SARS-CoV-2 is a relatively recent occurrence. Thus, bnAbs directed towards HCoVs are likely to be the next big step in human preparedness and counteraction to pandemic coronaviruses. Despite vaccines availability, SARS-CoV-2 continues to mutate and new HCoVs may emerge in the next years that may escape SARS-CoV-2-directed vaccines. Therefore, the search for HCoV bnAbs, their therapeutic and diagnostic implementation, and the study of their molecular targets for the development of a universal vaccine should continue in the interest of global public health preparedness. A perspective is proposed here for a strategy to pursue the search for HCoV bnAbs using state of the art optical fiber biosensors, followed by the possible implementation of such bnAbs to devise universal coronavirus detectors.

## Implementing Different Technologies to Discover HCoV bnAbs

**FIGURE 1:**
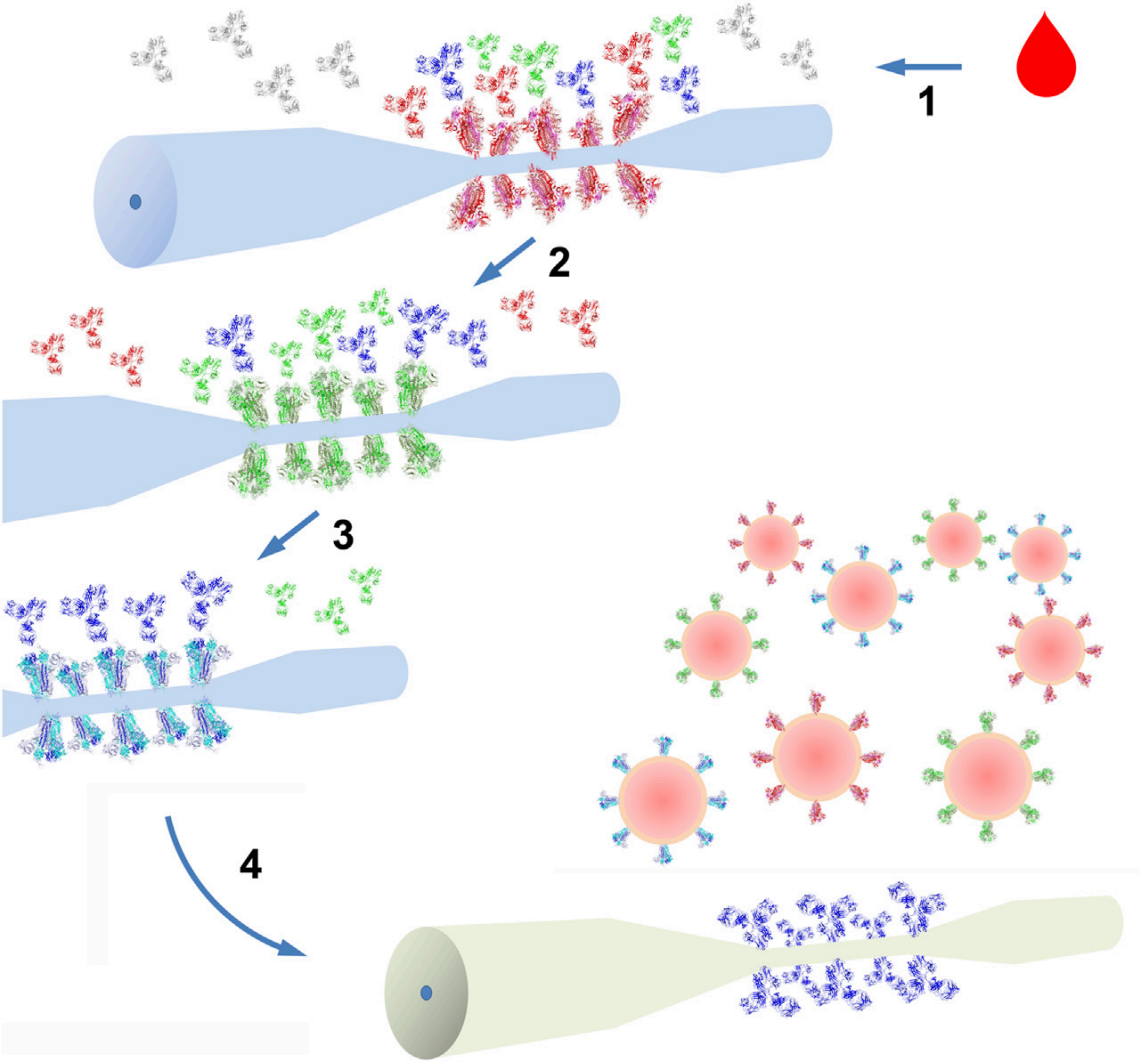
Schematics of HCoV bnAbs discovery and implementation. Blood samples from COVID-19 survivors (1) undergoes testing for SARS-CoV-2 spike antibody (red, green and blue) specificity (irrelevant antibodies are represented in grey). Antibody purification and testing for SARS-CoV spike antibody (green and blue) specificity is the following step (2). Next, a second antibody purification step and a final testing for MESR-CoV spike (3) can reveal brAbs (blue). After single B cell isolation from survivors’ blood, antibody production and testing for broad neutralization, brAbs and bnAbs (4) can be used to devise optical fiber biosensors for the universal detection of HCoVs (red, green and blue spiked viruses). Optical fibers represented are eTFBG, as an example of biosensor technology. Protein 3D structures were generated using PyMOL and represented in ribbon: SARS-CoV-2 spike trimers (red) are from PDB entry 6VYB, SARS-CoV (green) from PDB entry 5XLR, MERS-CoV (blue) from PDB entry 5 × 5F and antibodies from PDB entry 1IGY (IgG1).

We propose here the functionalization of three optical fiber biosensors with different pandemic betacoronaviruses’ trimeric spikes, namely from SARS-CoV-2, SARS-CoV and MERS-CoV. The strategy consists in the parallel and serial detection of antibodies specific for the different spikes. Blood from COVD-19 survivors would be immediately tested in parallel on the three biosensors, in order to inform about the presence of antibodies recognizing all three HCoVs’ spikes. Those rare samples that present detection by the three biosensors will then be subjected to serial detection by the same biosensors in the SARS-CoV-2, SARS-CoV and MERS-CoV sequence (Figure 1). The small sample quantity required to attain optical fiber biosensing is a crucial element in this process. Between the SARS-CoV-2 and SARS-CoV detections, antibodies will be purified by SARS-CoV-2 spike affinity chromatography. In this way, only antibodies that recognize SARS-CoV-2 spike will be tested for SARS-CoV recognition on the second biosensor, thus attesting their simultaneous recognition of both HCoVs. Only a portion of the samples are expected to contain antibodies recognizing both spikes. In a similar final step, following a SARS-CoV-2 spike affinity purification, antibodies will be tested for MERS-CoV spike detection, hence possibly proving the simultaneous detection of the three HCoV spikes. Blood from individuals whose samples present MERS-CoV detection after this serial detection-purification scheme will be assayed to isolate single B cells that in turn will be screened for MERS-CoV-specific antibodies, a technology largely implemented to isolate monoclonal antibodies from infectious diseases survivors (Lanzavecchia et al., 2007). Antibodies obtained in this way will be tested to recapitulate their broad recognition in the three HCoV spike biosensors. Subsequently, these broadly recognizing antibodies (brAbs) can finally be tested for HCoV neutralization and those presenting concomitant neutralization of SARS-CoV, SARS-CoV-2 and MERS-CoV will prove their status as bnAbs. Clearly, the discovery of HCoV bnAbs would open a large array of possibilities, ranging from their use for passive immunization in any HCoV outbreak to their molecular characterization to study vulnerable HCoV conserved sites in the spike, an essential informative instrument in the development of a universal vaccine for HCoV. In the case of absence of broad neutralization by brAbs, they will still represent an invaluable tool for the assembly of HCoV detectors.

## Devising HCoV Optical Fiber Detectors

HCoV brAbs and bnAbs obtained with the strategy described above would be identically useful for the assembly of a universal HCoV detector based on optical fiber biosensing (Figure 1). Using virus-specific antibodies, optical fiber biosensors specific for each of the three pandemic HCoVs can be developed, providing a specific detection signal for each different virus. The advantage of optical fiber biosensors is their suitability for multiplexing: multiple biosensors could be deployed either within the same optical fiber or on different fibers. Optical fibers can be functionalized by coating with metal layer such as gold or by forming a silane layer directly on the fiber surface. The silane layer is straightforward and the most common method of glass optical fiber modification. Therefore, bioreceptors can be immobilized via coupling with chemicals such as glutaraldehyde (either directly or via a linker proteins) and blocking the unreacted surface with bovine serum albumin (Luo et al., 2015; Srinivasan et al., 2017; Loyez et al., 2018; Sypabekova et al., 2019). The modified sensor could then be tested for its binding to different analytes (in buffer and spiked clinical samples). The development of optical fiber biosensors capable to detect HCoVs with high specificity and sensitivity would have a manifold importance, from diagnostic purposes to environmental monitoring. Optical fiber technology has been extensively used for telecommunication, therefore much of the logistics for deployment can be adopted for medical point of care but also to secure perimeters of sensitive building and areas from biological threats deriving from natural origins or deliberate release. Our group is presently working on a similar approach to develop portable optical fiber biosensors to detect poxviruses, among which the smallpox pandemic-causing *Variola virus* is a Category A bio-warfare/bioterrorism agent.

## Optical Fiber Biosensors: Technological Aspects

Over the last decades, biosensors witnessed increasing interest. The vast majority of biosensors, including electrochemical, piezo-electrical and optical were claimed to have extremely sensitive and fast detection at reduced cost, however there are still no commercially available biosensors that could be widely used during pandemics such as COVID-19. The key characteristics of the “ideal biosensor” to be taken into consideration for an effective use in a pandemic are: high selectivity and sensitivity, rapid response time, multiplexing, multi-mode sensing, disposability, long shelf life, ease to use, cost effectiveness, mass manufacturing, autonomy and connectivity to central health care systems (Bhalla et al., 2020). Amongst all types of biosensors, electrochemical biosensors draw a considerable attention due to their reduced limit of detection, cheap manufacturing costs and ease of surface functionalization. However, the signal from such sensors can be affected by the frequency variation of the electromagnetic interference (EMI). Another limitation of such sensors is the difficulty in obtaining a consistent signal while testing a complex biological media as well as the inability to be used in difficult to reach environments. Another type of widely studied biosensor is based on optical fibers, including grating-assisted (Lao et al., 2019; Sypabekova et al., 2019) and grating-free optical fibers (Sypabekova et al., 2020), photonic crystals (Gharsallah et al., 2018), resonators (Pongruengkiat and Pechprasarn, 2017) and interferometers (Liu et al., 2013) The first interest in optical fiber-based biosensors started with medical applications in the field of endoscopy and laser (Socorro-Leránoz et al., 2019). Since then, several studies showed that, by modifying the fiber structure (grating inscription, nanoparticle deposition, etching and tapering), it is also possible to detect binding events around the fiber. Detection is then transduced by a guided light inside the fiber and the light intensity, or the wavelength shift changes are used to report the results. In addition to the above mentioned “ideal biosensor” characteristics, optical fiber-based biosensors are biocompatible, capable of working in hazardous environments and complex biological media (Loyez et al., 2019), and unaffected by EMI unlike electrochemical biosensors (Socorro-Leránoz et al., 2019).

**FIGURE 2:**
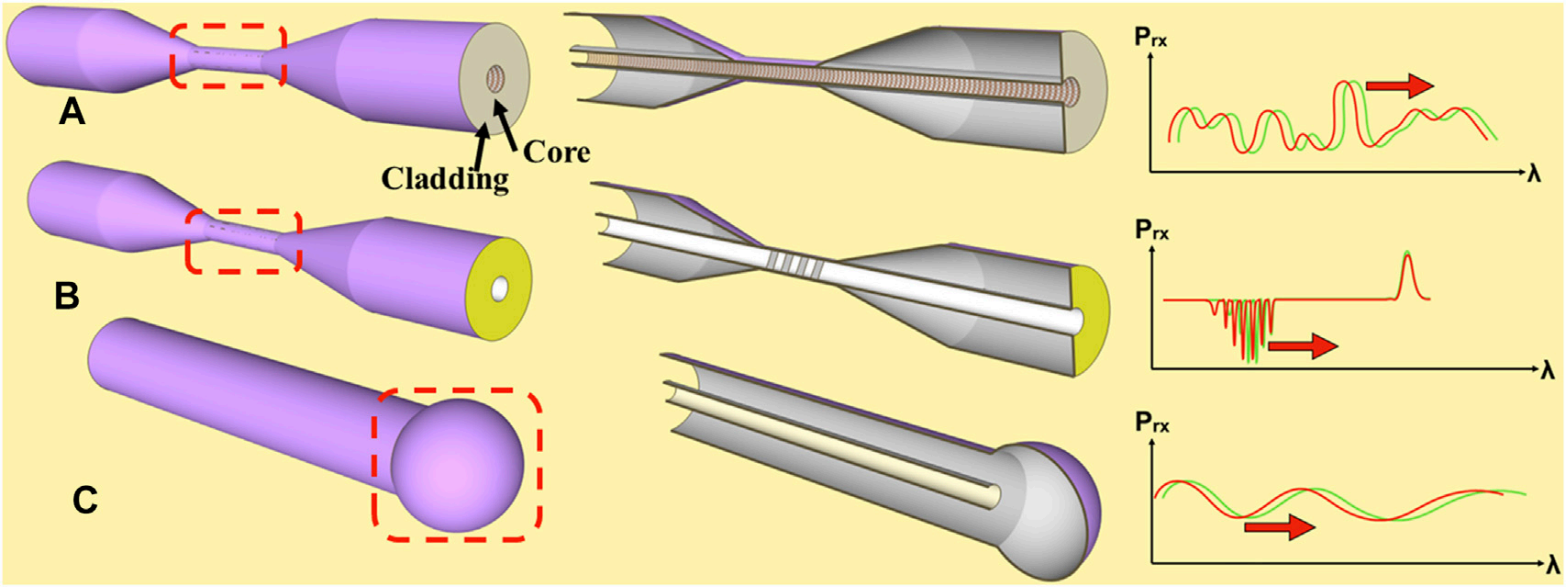
Schematic diagram of optical fiber based biosensors. **(A)** MgO nanoparticle doped-fiber, **(B)** eTFBG and **(C)** ball resonator, each biosensor has its corresponding cross section (middle) and the respective spectrum (right). Highlighted in red (dotted rectangle) is the sensing region.

Optical fiber-based biosensors can make use of standard telecommunication fibers, massively used in other fields, and made of two components: cladding (diameter 125 µm) and core (8–9 µm) (Figure 2). The light usually travels through the fiber core at total internal reflection mode without any propagation loss. For biosensing applications, the internal structure of the standard telecommunication fiber is usually modified. This modification is needed for the propagated light to escape the total internal reflection mode so that in can interact with the fiber surrounding at specific sensing points. Once the light interacts with the fiber surrounding media, the signal (e.g., wavelength change) becomes extremely sensitive to the surrounding refractive index (RI) change (i.e., due to the biological binding event). Therefore, in an optical fiber biosensor configuration, the surface of the fiber is modified with a receptor specific for a certain type of ligand. Up to now, DNA aptamers (Lepinay et al., 2014), antibodies (Sridevi et al., 2015), enzymes (Chen et al., 2019), other proteins (Voisin et al., 2014) and cellular adhesive components (Shevchenko et al., 2014) have been used in optical fiber biosensors. The detection of proteins (Ribaut et al., 2017), small molecules (Hu et al., 2018), DNA (Candiani et al., 2012), viruses (Luo et al., 2018), bacteria (Srinivasan et al., 2017) and human cells (Loyez et al., 2020) have been studied using various types of optical fiber.

Among the different optical fiber biosensors, we focus here on three distinct configurations, namely, etched tilted fiber Bragg grating (eTFBG), nanoparticle doped-fibers and ball-resonators, due to their improved sensing characteristics and easy fabrication steps. These sensors are entirely based on single-mode fibers and use telecom-grade analyzers, that detect spectral variations equal or lower than 1 pm and power levels with 0.01 dB precision and provide outstanding performance ratings. The ball-resonator has the fastest fabrication method so far, while the reflector-less biosensors have the potential to be the only manufacturing-less method by engineering the fibers; the eTFBG has the easiest spectral detection method unlike other sensors (such as plasmonic sensors that require additional hardware, U-bent fibers that cannot work as *in situ* probes, or long period gratings that are hard to manufacture and extremely sensitive to fiber bending). In our perspective, we single out three technologies that have been proven, both in silico and *in vitro*, and that have the same level of depth in terms of testing and reporting of the other sensors based on single-mode fibers. All the proposed sensors are based on a single-step or two-step manufacturing which can be easily implemented in a lab to be a disposable sensor, with parallel/multiplexing option.

## Etched TFBG

TFBG are extensively studied for biomolecular detection. They are made of standard single mode fibers in which gratings are inscribed inside the fiber core at a tilted angle (Figure 2B) through the phase mask or a femtosecond laser. The sensing principle in TFBF is dependent on the excitation of the forward propagating cladding modes that are able to reach the region between the cladding and the surrounding medium. The resulting signal or response is based on wavelength shift and an amplitude change upon the change in the surrounding RI. In many works, TFBG’s have been used together with the generation of the surface plasmon resonance (SPR) phenomenon in which the thin metal layer was deposited over the sensing region. Overall, this method increases the bulk sensitivity (Chiavaioli et al., 2017). The application of metal nanoparticles on the surface of the TBFG was also exploited for the generation of localized SPR and showed further increase in the sensitivity of the device (Shao et al., 2011; Lao et al., 2019). Although the SPR or localized SPR generation on the surface of the TFBG sensing region provided increased sensitivity, it involved an additional step in the fabrication process which requires cleaning and precisely controlled homogenous metal deposition and extra instrumentation (Suzuki et al., 2008). To avoid such processes, several reports used the SPR-less detection without additional fabrication steps (Bekmurzayeva et al., 2018). The surface chemistry based on covalent immobilization of the receptors on non-metallic coated optical fibers showed a high quality spectrum resonances changes and thus the possibility to generate optical fiber biosensors without the use of thin metal layer deposition (Loyez et al., 2018). The hunt for a device with increased sensitivity resulted in the production of etched TFBGs in which the cladding portion of the optical fiber was chemically depleted. Chemical etching is commonly used in mass production of electronic circuitry and therefore considered as a compatible technology with high-volume manufacturing (Spierings, 1993). The combination of the fiber photo inscription with wet chemical etching provided TFBG-based biosensors with enhanced sensitivity as compared to other TFBG configurations. The estimated theoretical limit of case protein-thrombin detection was 0.075 nM, a value about one order of magnitude lower than other reported fiber gratings for thrombin detection (Sypabekova et al., 2019). This particular set up showed that etched TFBG-based biosensors could outperform the grating-based configurations reported so far.

## Nanoparticle Doped-Fibers

Recent studies reported an alternative to the grating inscribed optical fibers named grating-free based optical fiber biosensors. Since gratings inside the fiber core were used to reflect the transmitted light, such grating-free sensors are also called reflector-less, capable of detecting the change in surrounding RI (Figure 2A). The detection in such devices is based on intrinsic reflection already present in the fiber and on processing the information and demodulation of the scattering power traces of backscattering fiber (Korganbayev et al., 2019) at wide range of RI. Such reflector-less devices eliminate the extra production steps such as grating inscription as well as fabrication of reflective gold mirrors at the tip of the fiber, and the use of external polarization controllers (Sypabekova et al., 2020) serving towards the reducing the production cost. The intrinsic property of such fibers includes the generation of the Rayleigh scattering that acts as the reflective element inside the fiber core. Optical fibers such as nanoparticle doped at the core generate a good Rayleigh scattering. Again, this type of fibers makes use of standard telecommunication fiber inside of which randomly sized nanoparticles are distributed along the entire fiber length, spanning from 1 cm up to several meters. Nanoparticle doped-fibers are usually produced by an established cost-effective and optimized chemical vapor deposition method (Sypabekova et al., 2018). The Rayleigh backscattered fibers scatter the light with a gain; hence they tend to scatter the light more than standard fibers. The other fabrication step involved in the production of nanoparticle doped-optical fiber biosensors is a chemical etching, the same method used for etched TFBG’s. The etching process in this case induces the scattering by the doped nanoparticles present in the fiber core and hence serves as an inductor for an increased Rayleigh scattering. The highest sensitivity reached using nanoparticles doped-fiber was 19.63 nm/RIU for RI from 1.3329 up to 1.37649 in a sensor functionalized with DNA aptamers to detect thrombin molecule (Sypabekova et al., 2020).

The major limitation of using etched sensors (both TFBG and nanoparticle doped-fibers) is the fragility of the fiber as the result of the etching process where the operational diameter of the sensing surface becomes extremely small and hence can be easily broken. To avoid this, a proper set up needs to be built to avoid physical disturbance and hence to perform the measurement without breaking the fiber.

## Ball-Resonators

A ball-resonator is a spherically shaped termination of an optical fiber, which acts as a multipath interferometer (Figure 2C). Unlike the previous method, ball-resonators have larger size (usually 100–600 μm diameter), which improves the mechanical stability of the probe and the robustness and offers a larger surface of interaction (Harun et al., 2013). The main advantage of the ball-resonators is their ease of fabrication. The tip-resonator can be obtained using a CO_2_ laser fiber splicer in a single step, collapsing two single-mode fibers (Kostovski et al., 2014). The process takes up to few seconds and is highly repeatable, forming interferometers with accurate size, ellipticity and low alignment errors. The ball-resonator uses the same fabrication principle of a spherical lens, used in near-field microscopy, but can be more easily interrogated by measuring the reflection spectrum of the device when the input light is fed from the resonator fiber input. The drawbacks of the ball-resonators are the low reflectivity, which requires a photodetector with excellent sensitivity (such as an optical backscatter analyzer), and the poor visibility of the spectral fringes, due to the weak interference phenomenon observed in the sphere between the multiple reflective paths. Nevertheless, such resonators show ellipticity of <0.5%, and enhanced sensitivity to the RI change in the range of 257.3–1,112.5 nm/RIU with diameter of 466–624 μm fabricated with a CO2 laser splicer (Shaimerdenova et al., 2020). Ball-resonators have been used for the detection of thrombin and CD44 with a detection limit at pM level (Ayupova et al., 2021; Bekmurzayeva et al., 2021).

## Discussion

Development of point of care devices where healthcare is more patient centered has become a global trend and contributed to the growth of more convenient and effective tests (St John and Price, 2014; Cui et al., 2016; Choi et al., 2018; Pang et al., 2018), including the development of biosensors for SARS-CoV-2. Detection of antibody response to SARS-CoV-2 infection can be used to confirm COVID-19 disease (Bhalla et al., 2020). Usually, antibodies specific for SARS-CoV-2 are detected in biochemical tests such as enzyme-linked immunosorbent assay (ELISA) using standard 96 well microtiter plates. ELISA requires several incubations and washing steps before the final readout. Biochemical tests present a number of pitfalls, including SARS-CoV-2 cross-reactivity by antibodies generated against other coronaviruses, low levels of antibodies in samples and lack of sensitivity (Lv et al., 2020). In addition, with the continuous emergence of new variants the efficacy of these sensors may be compromised. Conversely, the search and use of bnAbs targeting the most conserved parts of the spike predicts a more reliable and prolonged detection.

We propose here the detection of both HCoV-specific antibodies and HCoVs, repurposing detection from disease diagnostics to antibody discovery and environmental detection of viruses, respectively. In parallel, we consider addressing the most important aspect of biosensors, sensitivity. By immobilizing the spike of SARS-CoV-2, SARS-CoV and MERS-CoV in a serial fiber optic biosensor detection set up, we envisage the identification of broadly recognizing (and possibly neutralizing) antibodies. Etched TFBGs, nanoparticle doped-fibers as well as ball-resonators can be used as RI sensors, prone for temperature compensation (Korganbayev et al., 2019) (Sypabekova et al., 2018). Once identified and characterized, bnAbs will add a formidable weapon to the fight of HCoV and set passive immunization preparedness for future pandemics. Interestingly, the same bnAbs can be used to assemble HCoV fiber optic detectors capable of sensing and revealing the presence of human pandemic coronaviruses in the environment. Therefore, this technology can be applied for multiple purposes, ranging from antibody discovery to virus detection and diagnosis, as well as the environmental detection of biological threats.

## Data Availability

The original contributions presented in the study are included in the article/Supplementary Material, further inquiries can be directed to the corresponding author.
